# High Risk of *Plasmodium vivax* Malaria Following Splenectomy in Papua, Indonesia

**DOI:** 10.1093/cid/ciy403

**Published:** 2018-05-16

**Authors:** Steven Kho, Benediktus Andries, Jeanne R Poespoprodjo, Robert J Commons, Putu A I Shanti, Enny Kenangalem, Nicholas M Douglas, Julie A Simpson, Paulus Sugiarto, Nicholas M Anstey, Ric N Price

**Affiliations:** 1 Global and Tropical Health Division, Menzies School of Health Research and Charles Darwin University, Darwin, Northern Territory, Australia; 2 Timika Malaria Research Program, Papuan Health and Community Development Foundation, Timika, Papua; 3 Rumah Sakit Umum Daerah Kabupaten Mimika, Timika, Papua; 4 Pediatric Research Office, Department of Pediatrics, University of Gadjah Mada, Yogyakarta, Indonesia; 5 Centre for Epidemiology and Biostatistics, Melbourne School of Population and Global Health, University of Melbourne, Victoria, Australia; 6 Rumah Sakit Mitra Masyarakat, Timika, Papua, Indonesia; 7 Center for Tropical Medicine and Global Health, Nuffield Department of Medicine, University of Oxford, United Kingdom

**Keywords:** malaria, vivax, falciparum, splenectomy, Indonesia

## Abstract

**Background:**

Splenectomy increases the risk of severe and fatal infections; however, the risk of *Plasmodium vivax* malaria is unknown. We quantified the *Plasmodium* species-specific risks of malaria and other outcomes following splenectomy in patients attending a hospital in Papua, Indonesia.

**Methods:**

Records of all patients attending Mitra-Masyarakat Hospital 2004–2013 were reviewed, identifying those who underwent splenectomy. Subsequent risks of specific clinical outcomes within 12 months for splenectomized patients were compared to nonsplenectomized patients from their first recorded hospital admission. In addition, patients splenectomized for trauma 2015–2016 were followed prospectively for 14 months.

**Results:**

Of the 10774 patients hospitalized during 2004–2013, 67 underwent splenectomy. Compared to nonsplenectomized inpatients, patients undergoing splenectomy had a 5-fold higher rate of malaria presentation within 12 months (adjusted hazard ratio [AHR] = 5.0 [95% confidence interval (CI): 3.4–7.3], *P* < .001). The AHR was 7.8 (95% CI: 5.0–12.3) for *P. vivax* and 3.0 (95% CI: 1.7–5.4) for *P. falciparum* (both *P* < .001). Splenectomized patients had greater risk of being hospitalized for any cause (AHR = 1.8 [95% CI: 1.0–3.0], *P* = .037) and diarrheal (AHR = 3.5 [95% CI: 1.3–9.6], *P* = .016). In the 14-month prospective cohort, 12 episodes of *P. vivax* and 6 episodes of *P. falciparum* were observed in 11 splenectomised patients.

**Conclusions:**

Splenectomy is associated with a high risk of malaria, greater for *P. vivax* than *P. falciparum*. Eradication of *P. vivax* hypnozoites using primaquine (radical cure) and subsequent malaria prophylaxis is warranted following splenectomy in malaria-endemic areas.

## Teaser

In Papua, splenectomized individuals have greater risk of malaria in the 12 months following splenectomy but not of mortality. Malaria risk was higher for *Plasmodium vivax* than *P. falciparum*. Early radical cure and prophylaxis are warranted in malaria endemic areas.

The spleen plays a vital role in host immunity and erythrocyte regulation. Reduced splenic function increases the risk of severe and fatal infections, particularly those from encapsulated bacteria, and parasites such as *Plasmodium* and *Babesia* [1–5]. Although patients undergoing splenectomy should be vaccinated and take antimicrobial prophylaxis to minimize these risks, these are rarely delivered in resource-poor settings.

Malaria remains the most important human parasitic disease, with approximately 3.4 billion people at risk [6]. The spleen is vital for immunity to *Plasmodium* species [1, 7–11]. Splenectomized patients are more susceptible to severe falciparum malaria and have a greater risk of hospitalization and mortality [12–14]. Studies of splenectomized individuals in malaria-endemic regions of Malawi and Papua New Guinea suggest a risk of malaria almost double that of nonsplenectomized controls [1, 2]. No studies have quantified the risk of vivax malaria after splenectomy. This is important given the recent recognition of *P. vivax* as a major cause of morbidity in malaria-endemic areas, including anemia and a risk of severe and fatal disease [15–17].

The aim of this study was to quantify the clinical consequences of splenectomy, particularly the species-specific risks of malaria, in individuals living in a malaria-endemic area in Indonesia.

## METHODS

Between April 2004 and December 2013, data were prospectively collected as part of routine surveillance of patients presenting to a referral hospital in Timika, Papua, Indonesia. A retrospective analysis of this data set quantified the comparative risk of malaria and other clinical outcomes in patients who had undergone splenectomy compared to non splenectomy patients. In addition, from 2015–2016, a group of splenectomized individuals were followed prospectively and the risks of malaria over a 14-month period quantified.

### Study Site

Timika is located in Mimika district, south-central Papua, Indonesia [17, 18]. Approximately 200000 people populate the area comprising highland-Papuans, lowland-Papuans and non-Papuan migrants [18]. Malaria transmission in Timika is high but unstable in lowland areas, with parasite prevalences of 13.9% and 38% by microscopy and polymerase chain reaction, respectively [19].

### Hospital and Malaria Treatment

The study was conducted at Mitra Masyarakat hospital (RSMM), which served 100% of malaria patients attending surrounding healthcare facilities before November 2008, and ~80% thereafter. The hospital has 110 inpatient beds, surgical theatres, and outpatient clinics. Approximately 1800 patients are reviewed each week. The hospital laboratory does not have bacteriology facilities.

Quinine and chloroquine were used as the first-line treatment of uncomplicated malaria due to any *Plasmodium* species until March 2006. Following this, treatment of uncomplicated malaria changed to dihydroartemisinin-piperaquine plus a 14-day course of primaquine for vivax malaria [20]. Post-splenectomy antimalarial and antibiotic prophylaxis was not hospital policy.

### Retrospective Data Collection

All patients are given a unique hospital record number (HRN) at their first hospital presentation, and all subsequent presentations can be linked to this HRN. Hospital clerks electronically record basic demographic information, mortality data, and the diagnoses given by the attending doctor (classified using the International Classification of Diseases) for each patient-presentation to hospital. Laboratory and pharmacy records are collected separately, with patients also identified by their HRN.

All inpatients and febrile outpatients are required to have a Giemsa-stained blood film checked for malaria by trained microscopists or a rapid-diagnostic-test. Previous quality-control of RSMM microscopy suggests an accuracy of malaria diagnosis of >90% [17].

### Identifying Splenectomy Patients

To identify all splenectomy patients accurately during the study period, surgery logbooks and registers from 2004 to 2013 were analyzed by 2 independent researchers (S. K. and B. A.) to identify individuals who may have had splenic surgery. Medical records of these individuals were then checked manually to confirm the diagnosis and reasons for surgery.

### Data Preparation

Clinical and laboratory data were merged into a single database using the HRN and date of presentation. All splenectomy patients were aged between 13 and 51 years, and none were pregnant at the time of splenectomy. The primary comparative group was therefore nonsplenectomy controls, identified from nonpregnant patients aged between 12 and 60 years on their first hospital admission for any reason other than splenic surgery. In a sensitivity analysis, clinical outcomes post-splenectomy were compared to outcomes in patients who were admitted for trauma who did not undergo splenectomy (search terms for nonsplenectomy trauma control in Supplementary File 1).

Malaria reinfection or relapse within 2 weeks of an initial infection is highly unlikely [21], and therefore multiple malaria presentations within 14 days were considered to represent a single episode. No splenectomy patients were infected or represented with *P. malariae* or *P. ovale*.

### Statistical Analyses

All statistical analyses were performed in SPSS v23. Baseline characteristics were presented for the splenectomized and nonsplenectomized patients and compared using the χ^2^ test. In the primary analysis, the risks of representation with any malaria, *P. falciparum* malaria and *P. vivax* malaria by 12 months were compared in splenectomized versus nonsplenectomized patients. Patients were assumed to have been present in Timika for the full 12 months from the date of their splenectomy or first hospital admission (for controls). Study follow-up was curtailed at 31 December 2013. Other outcomes compared included risk of admission with other clinical outcomes (pneumonia, diarrheal illness, sepsis, cellulitis, tuberculosis, and urinary tract infections), any hospital representation, any admission, and death. The incidence of these outcomes was calculated from the cumulative number of episodes observed over 12 months or until the last day of follow-up and reported as a rate “per thousand patient-years.” Analyses of the time- to-first-event were calculated by survival analysis and compared using Kaplan-Meier and Cox regression models. Potential confounders included in the multivariable Cox models were age (<15 years, ≥15–60 years), sex, ethnicity (highland-Papuan, lowland-Papuan, non-Papuan), and presence and species of parasitemia at the time of initial admission. To control for changes in background malaria endemicity, multivariable Cox models were stratified by year of splenectomy or year of first hospital admission for controls. In the sensitivity analysis, comparisons were repeated between splenectomy patients and nonsplenectomy trauma controls.

### Prospective Cohort

In a complementary prospective study, 11 patients undergoing splenectomy at an adjacent hospital (Rumah Sakit Umum Daerah [RSUD]) between July 2015 and November 2016 were followed from their day of splenectomy for 14 months. Malaria encounters were recorded by monitoring visits to healthcare facilities and conducting monthly interviews. Patients were checked for peripheral parasitemia on the day of surgery and were treated according to local treatment guidelines. Data from these patients are presented using descriptive statistics only.

### Ethical Approval

This study was approved by the Ethics Committees of the University of Gadjah Mada (KE/FK/544/EC, January 2015), Indonesia, and Menzies School of Health Research, Australia (10.1397, September 2014). In the retrospective cohort, hospital records were anonymized with informed consent not requested because data were collected as part of routine hospital surveillance. Informed consent was obtained in the prospective cohort.

## RESULTS

**Figure 1.**
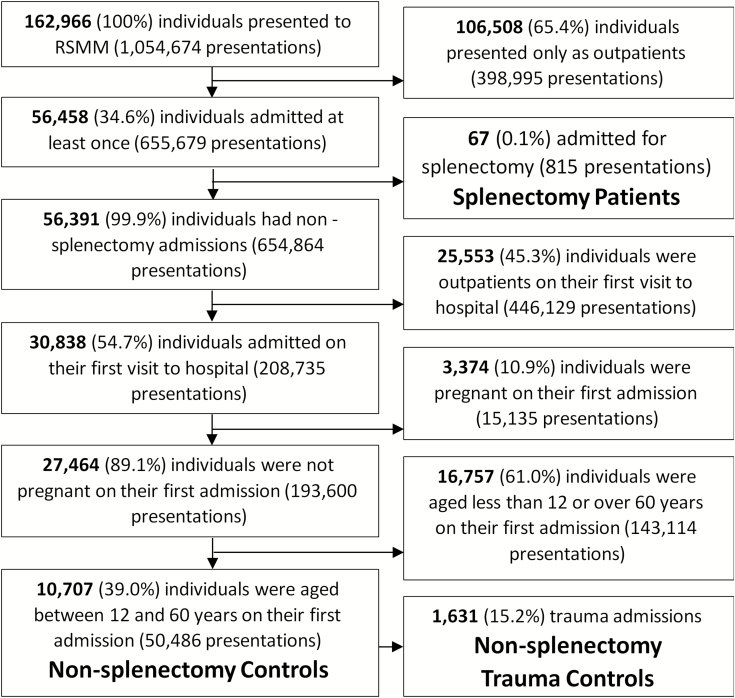
Flow diagram of data merging process and exclusion criteria. Of the 162966 patients attending the main hospital between 2004 and 2013 in the Mimika district of southern Papua, Indonesia, 67 underwent splenectomy and 10707 were considered nonsplenectomy controls, of which 1631 were controls admitted following trauma-related diagnoses. Abbreviation: RSMM, Mitra Masyarakat Hospital.

Between April 2004 and December 2013, 162966 patients attended RSMM hospital of whom 56458 (34.6%) were admitted at least once and 67 patients underwent splenectomy (Figure 1). A total of 10707 non-pregnant patients aged 12–60 years admitted on their first encounter to hospital did not have a splenectomy, of whom 1631 (15.2%) presented with a trauma-related diagnosis (Figure 1).

### Baseline Characteristics

Of the 67 splenectomized patients, 43 (64.2%) were male, 44 (65.7%) were highland-Papuans, and 4 (6.0%) were lowland-Papuans; Table 1. The median age at the time of splenectomy was 25.4 years (range 13.7–51). The number of splenectomy patients per year varied with the highest number recorded in 2012. Fifty patients (74.6%) were splenectomized due to traumatic injury; Supplementary Table 1.

**Table 1.** Baseline Characteristics of Splenectomy Patients and Nonsplenectomy Controls

Variable	Splenectomy Patients	Nonsplenectomy Controls	*P*	Total	Nonsplenectomy Trauma Controls^a^	*P*
	No. (%)	No. (%)		No. (%)	No. (%)	
Sex						
Male	43 (64.2)	6558 (61.2)	.624	6601 (61.3)	1254 (76.9)	.016
Female	24 (35.8)	4149 (38.8)		4173 (38.7)	377 (23.1)	
Ethnicity^b^						
Non-Papuan	19 (28.3)	2745 (25.7)	.091	2764 (25.7)	482 (29.6)	.002
Highland-Papuan	44 (65.7)	6262 (58.6)		6306 (58.7)	780 (47.9)	
Lowland-Papuan	4 (6.0)	1678 (15.7)		1682 (15.6)	366 (22.5)	
Age, years						
Median [Range]	25.4 [13.7–51.0]	26.8 [12.0–60.0]		26.8 [12.0–60.0]	25.8 [12.0–60.0]	
12 to <15	1 (1.5)	543 (5.1)	.182	544 (5.0)	74 (4.5)	.235
≥15 to 60	66 (98.5)	10164 (94.9)		10230 (95.0)	1557 (95.5)	
Parasitemia^c^						
Negative	54 (80.6)	5936 (55.8)	<.001	5990 (55.9)	1484 (91.3)	.001
*P. falciparum*	10 (14.9)	3291 (30.9)		3301 (30.8)	75 (4.6)	
*P. vivax*	2 (3.0)	812 (7.6)		814 (7.6)	60 (3.7)	
Mixed infections	1 (1.5)	610 (5.7)		611 (5.7)	7 (0.4)	
Year						
2004	6 (9.0)	1462 (13.7)		1468 (13.6)	155 (9.5)	
2005	7 (10.4)	1540 (14.4)		1547 (14.4)	203 (12.5)	
2006	6 (9.0)	1484 (13.9)		1490 (13.8)	242 (14.8)	
2007	8 (11.9)	1481 (13.8)		1489 (13.8)	190 (11.6)	
2008	8 (11.9)	1075 (10.0)		1083 (10.1)	184 (11.3)	
2009	3 (4.5)	885 (8.3)		888 (8.3)	137 (8.4)	
2010	4 (6.0)	668 (6.2)		672 (6.2)	102 (6.3)	
2011	5 (7.5)	556 (5.2)		561 (5.2)	114 (7.0)	
2012	15 (22.3)	534 (5.0)		549 (5.1)	111 (6.8)	
2013	5 (7.5)	1022 (9.5)		1027 (9.5)	193 (11.8)	
Total	67 (100)	10707 (100)		10774 (100)	1631 (100)	

*P* values from χ^2^ test of control group versus splenectomy patients.

^a^Subgroup of patients from non-splenectomy controls.

^b^Data missing in 22 nonsplenectomy controls (3 trauma).

^c^58 nonsplenectomy controls with *P. malariae* or *P. ovale* infection omitted from analysis (5 trauma).

The nonsplenectomized patients had similar demographics to that of the cases, with a median age at first admission of 26.8 (range 12–60; Table 1). The number of nonsplenectomy controls per year peaked in 2005 and declined thereafter. Thirteen splenectomy patients (19.4%) had peripheral parasitemia at the time of surgery compared to 4713 patients (44.3%) at the time of admission in the nonsplenectomy control group (Table 1).

### Risk of Representation With Malaria

In total, 56.1% (6041/10774) of patients represented to hospital within 4 years following initial presentation, of whom 86.2% (5208/6041) did so within 12 months. The overall risk of representing with any malaria within 12 months was 13.5% (95% confidence interval [CI]: 12.9–14.2) and was greater in females compared to males (hazard ratio [HR] = 1.3 [95% CI: 1.2–1.5], *P* < .001), children compared to adults (HR = 1.4 [95% CI: 1.1–1.7], *P* = .001), and highlanders compared to non-Papuans (HR = 3.4 [95% CI: 2.9–4.0], *P* < .001). The risk of representing with malaria was also elevated in those presenting initially with malaria and this was apparent for all species; Table 2.

**Table 2.** Baseline Risk Factors for Representing With Any Malaria, *P. falciparum*, or *P. vivax* Within 12 months

Outcome	Baseline Risk Factor	Prevalence of Outcome (n/N)	Univariable Analysis		Multivariable Analysis^a^	
			HR [95% CI]	*P*	AHR [95% CI]	*P*
**Any malaria**	**Splenectomy**					
	No	13.3% (1426/10707)	Reference		Reference	
	Yes	43.3% (29/67)	4.3 [3.0–6.2]	<.001	5.0 [3.4–7.3]	<.001
	**Sex**					
	Male	12.2% (803/6601)	Reference		Reference	
	Female	15.6% (652/4173)	1.3 [1.2–1.5]	<.001	1.3 [1.1–1.4]	<.001
	**Age, years**					
	≥15 to 60	13.2% (1353/10230)	Reference		Reference	
	12 to 14	18.8% (102/544)	1.4 [1.1–1.7]	.001	1.2 [1.0–1.5]	.098
	**Ethnicity**					
	Non-Papuan	5.8% (160/2764)	Reference		Reference	
	Highland-Papuan	19.2% (1213/6306)	3.4 [2.9–4.0]	<.001	3.4 [2.8–4.0]	<.001
	Lowland-Papuan	11.9% (81/1682)	0.8 [0.6–1.1]	.122	1.0 [0.8–1.3]	.979
	**Parasitemia**					
	Negative	8.8% (530/5990)	Reference		Reference	
	*P. falciparum*	18.4% (607/3301)	2.1 [1.8–2.3]	<.001	1.8 [1.6–2.0]	<.001
	*P. vivax*	20.8% (169/814)	2.4 [2.0–2.8]	<.001	2.4 [2.1–2.9]	<.001
	Mixed infections	22.6% (138/611)	2.6 [2.2–3.2]	<.001	2.2 [1.8–2.6]	<.001
***P. falciparum***	**Splenectomy**					
	No	8.4% (901/10707)	Reference		Reference	
	Yes	17.9% (12/67)	2.3 [1.3–4.0]	.005	3.0 [1.7–5.4]	<.001
	**Sex**					
	Male	7.8% (518/6601)	Reference		Reference	
	Female	9.5% (395/4173)	1.2 [1.1–1.4]	.002	1.2 [1.0–1.3]	.016
	**Age, years**					
	≥15 to 60	8.3% (847/10230)	Reference		Reference	
	12 to 14	12.1% (66/544)	1.4 [1.1–1.8]	.006	1.2 [0.9–1.5]	.211
	**Ethnicity**					
	Non-Papuan	3.0% (84/2764)	Reference		Reference	
	Highland-Papuan	12.2% (770/6306)	4.0 [3.2–5.0]	<.001	3.7 [2.9–4.7]	<.001
	Lowland-Papuan	3.4% (58/1682)	1.1 [0.8–1.6]	.543	1.3 [0.9–1.8]	.119
	**Parasitemia**					
	Negative	5.5% (331/5990)	Reference		Reference	
	*P. falciparum*	12.4% (408/3301)	2.2 [1.9–2.5]	<.001	1.8 [1.6–2.1]	<.001
	*P. vivax*	11.2% (91/814)	2.0 [1.6–2.5]	<.001	2.0 [1.6–2.5]	<.001
	Mixed infections	12.3% (75/611)	2.2 [1.7–2.8]	<.001	1.8 [1.4–2.3]	<.001
***P. vivax***	**Splenectomy**					
	No	4.9% (523/10707)	Reference		Reference	
	Yes	31.3% (21/67)	7.7 [5.0–11.9]	<.001	7.8 [5.0–12.3]	<.001
	**Sex**					
	Male	4.1% (269/6601)	Reference		Reference	
	Female	6.6% (275/4173)	1.6 [1.4–1.9]	<.001	1.5 [1.3–1.8]	<.001
	**Age, years**					
	≥15 to 60	4.9% (505/10230)	Reference		Reference	
	12 to 14	7.2% (39/544)	1.4 [1.0–1.9]	.044	1.2 [0.9–1.7]	.224
	**Ethnicity**					
	Non-Papuan	2.6% (72/2764)	Reference		Reference	
	Highland-Papuan	7.2% (452/6306)	2.7 [2.1–3.4]	<.001	2.8 [2.2–3.6]	<.001
	Lowland-Papuan	1.2% (20/1682)	0.4 [0.3–0.7]	.001	0.6 [0.4–1.0]	.041
	**Parasitemia**					
	Negative	2.9% (173/5990)	Reference		Reference	
	*P. falciparum*	6.5% (214/3301)	2.1 [1.7–2.6]	<.001	2.0 [1.6–2.5]	<.001
	*P. vivax*	11.9% (97/814)	4.1 [3.2–5.2]	<.001	4.0 [3.1–5.1]	<.001
	Mixed infections	9.3% (57/611)	3.2 [2.4–4.3]	<.001	2.6 [1.9–3.5]	<.001

Abbreviations: AHR, adjusted hazard ratio; CI, confidence interval; HR, hazard ratio.

^a^Cox model stratified by year of splenectomy or 1st hospital admission (2004–2013).

**Figure 2.**
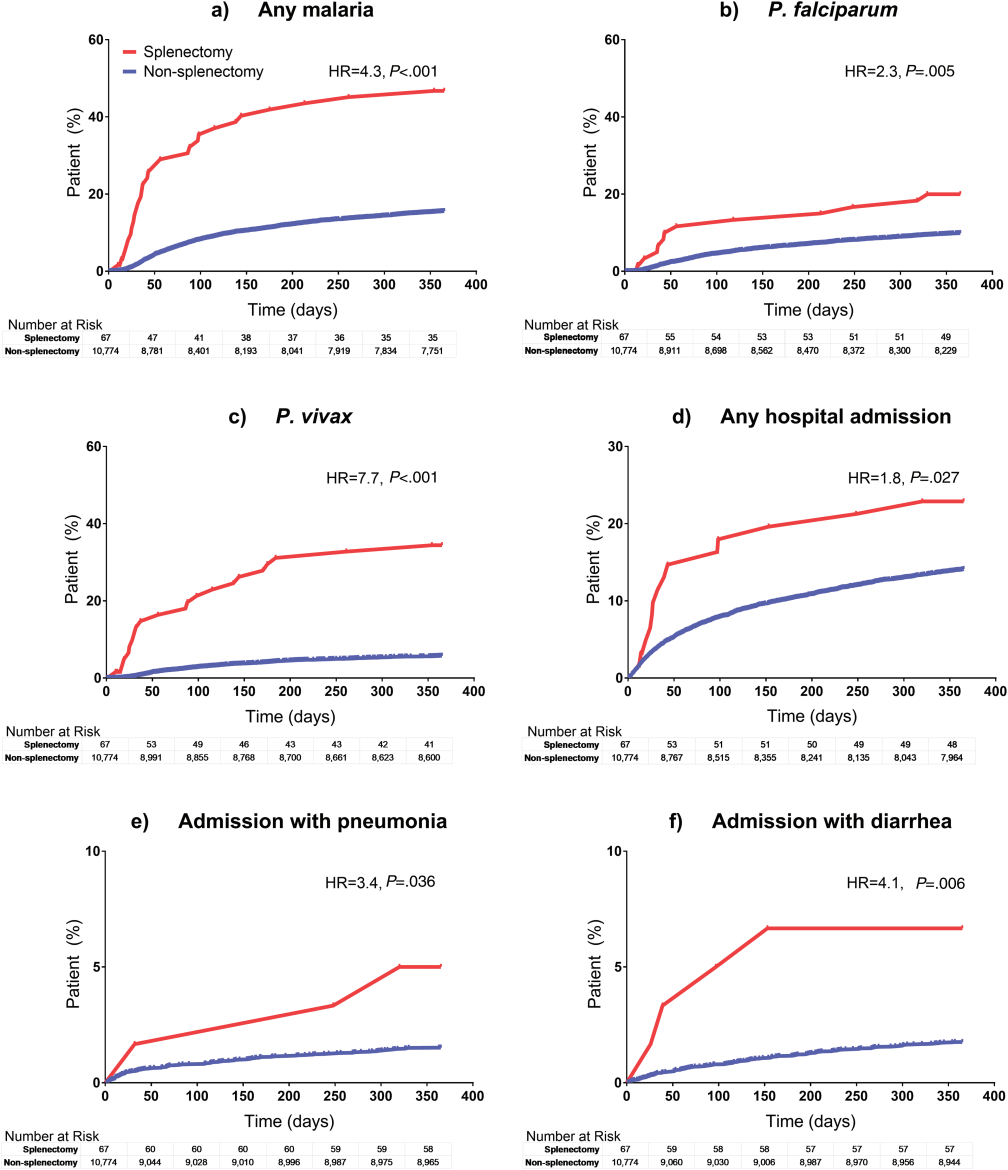
Kaplan-Meier survival curves showing time (in days) to first presentation with (*a*) any malaria, (*b*) falciparum malaria, and (*c*) vivax malaria, and time to first hospital admission with (*d*) any cause, (*e*) pneumonia, and (*f*) diarrhea, within 12 months of splenectomy (red), versus non-splenectomy controls (blue) in southern Papua, Indonesia. Univariable HRs (95% CI) and *P*-values are presented. The number of patients at risk at each time point is indicated below each graph. Abbreviations: CI, confidence interval; HR, hazard ratio.

Overall 43.3% (29/67) of splenectomized patients represented with malaria within 12 months of surgery compared to 13.3% (1426/10707) of those in the control group (HR = 4.3 [95% CI: 3.0–6.2], *P* < .001; Table 2 and Figure 2a). After categorizing malaria by species, 17.9% (12/67) represented with *P. falciparum* and 31.3% (21/67) with *P. vivax* within 12 months of splenectomy compared to 8.4% (901/10707) and 4.9% (523/10707) in the control group, respectively (Figure 2b and 2c). Of the 67 splenectomized patients, 10 (14.9%) had malaria in the 12 months preceding splenectomy compared to 29 (43.3%) in the 12 months following splenectomy (odds ratio [OR] = 7.3 [95% CI: 2.2–24.5], *P* < .001). The risk of malaria was similar in the subgroup of 50 patients splenectomized due to trauma (OR = 9.0 [95% CI: 2.1–38.8], *P* < .001). Having detectable parasitemia at initial presentation was a risk factor for representing with any malaria in the control group (HR = 2.3 [95% CI: 2.0–2.5], *P* < .001) but not in the splenectomy group (HR = 1.4 [95% CI: 0.5–3.6], *P* = .516). In total, 70.0% (20/29) of the first malaria episodes post-splenectomy occurred within the first 3 months, compared to 48.8% (696/1426 in controls; *P* = .032).

After controlling for confounding factors, splenectomy was a significant risk factor for hospital representation with any malaria (adjusted hazard ratio [AHR] = 5.0 [95% CI: 3.4–7.3], *P* < .001), and this was more apparent with *P. vivax* (AHR = 7.8 [95% CI: 5.0–12.3], *P* < .001) compared to *P. falciparum* (AHR = 3.0 [95% CI: 1.7–5.4], *P* < .001; Table 2). Of the 29 splenectomy patients representing with malaria within 1 year, 34.5% (10/29) had more than 1 episode of malaria, including 50% (4/8) of those with *P. falciparum*, 30% (6/19) of those with *P. vivax* and 0% (0/2) of those with mixed species infections.

The incidence of malaria was 699 (95% CI: 506–942) per thousand patient-years in splenectomy patients compared to 222 (95% CI: 213–232) per thousand patient-years in the nonsplenectomy controls (rate ratio [RR] = 3.1 [95% CI: 2.3–4.3], *P* < .001; Table 3). The rate of malaria associated with splenectomy was significantly higher for *P. vivax* (RR = 4.5 [95% CI: 3.1–6.7], *P* < .001) compared to that for *P. falciparum* (RR = 2.2 [95% CI: 1.4–3.5], *P* = .001; Table 3). In splenectomy patients, the incidence of malaria was 236 (95% CI: 132–389) per thousand patient-years in the 12 months presplenectomy compared to 699 (95% CI: 506–942) per thousand patient-years post-splenectomy (RR = 3.0 [95% CI: 1.6–5.7], *P* < .001; Table 3).

**Table 3.** Incidence Rate and Rate Ratio of Malaria Episodes and Hospital Representations and Admissions Over 12-Months Follow-up, Comparing Splenectomy Patients to Controls, and Comparing 12-Month Rate Pre- and Post-splenectomy

Outcomes	Incidence Rate per 1000 Patient-Years [95% CI]			12 Months Before vs 12 Months After Splenectomy		Splenectomy Patients vs Nonsplenectomy Controls	
	Splenectomy Patients 12 Months Before	Splenectomy Patients 12 Months After	Nonsplenectomy Controls 12 Months After	Rate Ratio [95% CI]	*P*	Rate Ratio [95% CI]	*P*
Any malaria	236 [132–389]	699 [506–942]	222 [213–232]	3.0 [1.6–5.7]	<.001	3.1 [2.3–4.3]	<.001
*P. falciparum*	220 [120–369]	325 [199–502]	146 [138–154]	1.5 [0.7–3.2]	0.133	2.2 [1.4–3.5]	.001
*P. vivax*	79 [26–183]	439 [289–639]	97 [91–103]	5.6 [2.1–8.6]	<.001	4.5 [3.1–6.7]	<.001
Any hospital representation	1556 [1265–1894]	4940 [4400–5528]	2145 [2116 –2174]	3.2 [2.5–4.0]	<.001	2.3 [2.1–2.6]	<.001
Any hospital admission	189 [97–329]	309 [186–482]	174 [166–183]	1.6 [0.8–3.7]	.092	1.8 [1.1–2.8]	.02

Abbreviation: CI, confidence interval.

### Risk of Other Morbidity and Mortality

The overall risk of representing at least once within 12 months was 82.1% (55/67) in splenectomy patients compared to 48.1% (5153/10707) in controls (AHR = 2.3 [95% CI: 1.8–3.0], *P* < .001; Table 4). During this period, splenectomy patients were at greater risk of being admitted to hospital for any cause (AHR = 1.8 [95% CI: 1.0–3.0], *P* = .037; Figure 2d), being admitted with pneumonia (AHR = 2.8 [95% CI: 0.9–8.8], *P* = .085; Figure 2e) or being admitted with diarrhea (AHR = 3.5 [95% CI: 1.3–9.6], *P* = .016; Figure 2f) but not being admitted for sepsis, cellulitis, tuberculosis, or urinary tract infection (Table 4 and Supplementary Tables 6–13). Twelve months after splenectomy, 6.0% (4/67) of splenectomy patients had died compared to 5.7% (613/10707) of controls (AHR = 0.8 [95% CI: 0.3–2.2], *P* = .671; Supplementary Table 5).

**Table 4.** Risk of Death, Representation, and Admission to Hospital With Different Clinical Outcomes Within 12 Months of Splenectomy Versus Nonsplenectomy Controls

Outcomes Observed Within 12 Months	Prevalence of Outcome (n/N)		Splenectomy Patients vs Non-splenectomy Controls			
	Splenectomy Patients	Nonsplenectomy Controls	Univariable Analysis		Multivariable Analysis^a^	
			HR [95% CI]	*P*	AHR [95% CI]	*P*
Death	6.0% (4/67)	5.7% (613/10707)	1.0 [0.4–2.8]	.951	0.8 [0.3–2.2]	.671
Any representation to hospital	82.1% (55/67)	48.1% (5153/10707)	2.7 [2.1–3.6]	<.001	2.3 [1.8–3.0]	<.001
Any admission to hospital	20.9% (14/67)	12.2% (1308/10707)	1.8 [1.1–3.1]	.027	1.8 [1.0–3.0]	.037
Admission with pneumonia	4.5% (3/67)	1.3% (138/10707)	3.4 [1.1–10.7]	.036	2.8 [0.9–8.8]	.085
Admission with diarrhea	6.0% (4/67)	1.5% (160/10707)	4.1 [1.5–11.0]	.006	3.5 [1.3–9.6]	.016
Admission with sepsis	1.5% (1/67)	0.4% (47/10707)	3.4 [0.5–24.4]	.230	2.6 [0.4–19.3]	.350
Admission with cellulitis	0% (0/67)	0.1% (13/10707)	0.0	.848	…	…
Admission with tuberculosis	0% (0/67)	2.5% (269/10707)	0.0	.380	…	…
Admission with UTI	3.0% (2/67)	1.3% (139/10707)	2.3 [0.6–9.2]	.250	2.3 [0.6–9.2]	.256

Abbreviations: AHR, adjusted hazard ratio; CI, confidence interval; HR, hazard ratio; UTI, urinary tract infection.

^a^Separate Cox model generated for each outcome, included co-variables, age, sex, ethnicity (non-Papuan, highland-Papuan, lowland-Papuan) and peripheral parasitemia (negative, *P. falciparum*, *P. vivax*, mixed infections) stratified by year of splenectomy or 1st hospital admission (2004–2013).

### Risk of Malaria in Patients With Trauma

Of the 10707 nonsplenectomy controls, 1631 (15.2%) patients were admitted due to trauma and were considered nonsplenectomy trauma controls (Figure 1). Baseline characteristics were similar between splenectomy and trauma patients (Table 1). When compared to nonsplenectomy trauma controls, the risk of malaria after splenectomy was even greater (AHR = 6.1 [95% CI: 3.9–9.3], *P* < .001; Supplementary Table 2).

### Malaria in a Cohort of 11 Patients Followed Prospectively From Splenectomy

Eleven patients underwent splenectomy at RSUD Hospital, all due to trauma, of which 9 (81.8%) were male, 6 (54.5%) were highland-Papuans, and 2 (18.2%) were lowland-Papuans (Supplementary Table 3). The median age at the time of splenectomy was 30 years (range 15–46). At the time of surgery, 4 had asymptomatic peripheral parasitemia by microscopy (1 *P. vivax* and 3 *P. falciparum*) and were given antimalarial treatment. In the 14 months following splenectomy, 8 of 11 (72.7%) patients returned with 18 episodes of symptomatic microscopically confirmed malaria: 8 (72.7%) patients with 12 episodes of *P. vivax* and 6 (54.5%) with 6 episodes of *P. falciparum* infection, none severe. Four of these 8 patients (50%) returned within 3 months of surgery, on day 8 (*P. vivax*), day 18 (*P. vivax*; having had asymptomatic vivax parasitemia on day 0), day 32 (*P. falciparum*; having had asymptomatic falciparum parasitemia on day 0) and month 3 (*P. vivax*), respectively. At 14 months of follow-up no patient had died.

## DISCUSSION

Our large observational study quantifies the risk of morbidity and mortality in splenectomized individuals living in a malaria-endemic region. Splenectomized patients were at 5-fold higher risk of malaria within 12 months of surgery, with the risk being far greater for *P. vivax* compared to *P. falciparum*. These retrospective findings were supported by data from a prospective cohort of 11 patients followed after splenectomy. Splenectomy patients were at increased risk of representation due to any cause, admission with diarrheal illness or pneumonia, but not mortality or other causes of infection.

Several case reports and small series highlight an increased risk of malaria after splenectomy; however, these reports are limited to no more than 33 splenectomized individuals [1, 2, 12–14, 22–26]. The current analysis was able to expand the risk analysis from an endemic area where a high number of individuals undergo splenectomy, mostly following trauma or road traffic injuries. Our study was also able to quantify, for the first time to our knowledge, the risk of vivax malaria after splenectomy and surprisingly noted that the risk was even greater than for falciparum malaria. Although our primary retrospective analysis was censored at 12 months, extending the period of follow-up to 4 years did not change the results meaningfully (Supplementary Table 4).

The greatest risk of malaria occurred in the first 3 months following splenectomy. In 2005 the incidence of malaria in Papua was approximately 876 per 1000 patient-years [18]. This comparatively low baseline incidence suggests that the rate of reinfection would have been relatively low and that episodes of malaria post-splenectomy were likely to be originating from low-level preexisting bloodstream infections. This is supported by 2 of the 4 patients with asymptomatic parasitemia in the prospective study developing clinical malaria within 32 days. Removal of the spleen impairs acquired immunity [1, 7, 9–11], and this may induce splenectomized patients with chronic, asymptomatic parasitemia to develop higher levels of parasitemia and become symptomatic [24, 25]. Alternatively, the splenectomy may result in displacement into, and/or multiplication within peripheral blood, of viable parasites that would otherwise preferentially accumulate as a hidden viable biomass in the spleen [27]. If *P. vivax* has a greater propensity for splenic pooling [27–29], this could then explain the higher risk of very early *P. vivax* recurrence compared to *P. falciparum.* Interestingly, after splenectomy a higher proportion of patients in the retrospective study had more than one episode of *P. falciparum* compared to *P. vivax*, suggesting that *P. vivax* attacks may have originated from preexisting infections or relapses that were then cleared, whereas *P. falciparum* recurrences were attributable to an increased susceptibility to new infections.

A third explanation for the increased risk of malaria is that attributable to trauma itself. The process of splenectomy is known to be followed by malaria in previously asymptomatic individuals [23–26]. Furthermore, there is an increased risk of falciparum malaria in trauma patients, a phenomenon known as postinjury malaria [30–32]. To address the confounding effect of trauma itself, a sensitivity analysis comparing splenectomized patients with control patients admitted due to trauma was performed. The risk of malaria was even higher in splenectomized patients, suggesting that trauma itself, at least in this population, is not a risk factor. We were unable to explore the effect of general surgery; however, earlier studies have shown that frequency of *P. falciparum* attacks or *P. vivax* relapses after surgery do not differ from the overall population or from pre-surgery [33–35]. Only one study reported a higher risk of *P. falciparum* in trauma patients undergoing primary surgery [31]. Only 11% of trauma patients are predicted to undergo surgery [36]; thus, surgery as a risk factor for malaria remains to be determined.

Previous studies have identified higher risk of sepsis including pneumonia following splenectomy [3, 4, 37, 38]. Although pneumonia was diagnosed in 141 patients, sepsis was recorded infrequently, a likely reflection of the lack of hospital microbiological facilities. Although splenectomized patients were at more than 2.5-fold greater risk of these outcomes, this did not reach statistical significance (*P* = .085 and .350, respectively). Our study was also underpowered to determine an increased risk of mortality following splenectomy, with only 4 (6.0%) patients known to have died within 12 months of splenectomy. The only outcome that reached statistical significance was a 3.5-fold increased risk of diarrheal admission. Salmonellosis is caused by encapsulated bacteria, and splenectomy patients are known to be at a high risk of disease [4, 39, 40]; however, without an underlying microbiological diagnosis we were unable to explore this further.

Our study has several limitations. Although our analysis was observational and free from coercive biases associated with many clinical study designs, our data are subject to the effects of residual confounding. Retrospective analyses were confined to RSMM presentations and did not consider presentations at other health facilities or emigration from the catchment area during the follow-up period. However, it is likely that this attrition bias would apply equally to the cases and controls. Furthermore, our findings were confirmed in the 11 patients followed prospectively. In the retrospective analysis, malaria occurring within 14 days of splenectomy was not captured and diagnosis by the attending physician was determined without microbiological testing.

In conclusion, splenectomized individuals in a malaria- endemic area are at 5-fold greater risk of malaria within 12 months of surgery, with 70% of events occurring in the first 3 months. The risk is greater for *P. vivax* than for *P. falciparum*. Current guidelines recommend that patients undergoing splenectomy should be offered vaccinations and lifelong prophylactic and standby antibiotics. Our study suggests that in malaria-endemic regions these individuals should also be offered early radical cure of malaria with an artemisinin combination therapy plus, in vivax-endemic regions, 14 days primaquine. In addition, malaria prophylaxis should be offered thereafter to prevent reinfection.

## Supplementary Data

Supplementary materials are available at *Clinical Infectious Diseases* online. Consisting of data provided by the authors to benefit the reader, the posted materials are not copyedited and are the sole responsibility of the authors, so questions or comments should be addressed to the corresponding author.

## Supplementary Material

Supplementary Material

## Notes

***Acknowledgments.*** We thank Yati Soenarto for support, Daniel Lampah, Leo Leonardo, Ruland Wandosa, and Hidar for assistance with patient record searches, staff and patients of Mitra Masyarakat Hospital, and participants in the prospective cohort.

***Funding.*** This work was supported by the Wellcome Trust (Senior Fellowship in Clinical Science to RNP, grant 200909) and the Australian National Health and Medical Research Council (NHMRC) (program grant 1037304 awarded to R. N. P. and N. M. A., and Practitioner and Research Fellowships [1042072 and 1135820] to N. M. A.). S. K. was supported by an Australian Government Postgraduate Award scholarship. J. R. P. was funded by a Wellcome Trust Training fellowship in Tropical Medicine (grant 099875). J. A. S. is supported by a NHMRC Research Fellowship. R. J. C. was supported by a postgraduate NHMRC scholarship and a RACP NHMRC Kincaid-Smith scholarship. The Timika Research Facility and Papuan Community Health Foundation are supported by the Australian Department of Foreign Affairs and Trade.

***Potential conflicts of interest.*** The authors declare no conflict of interest. All authors have submitted the ICMJE Form for Disclosure of Potential Conflicts of Interest. Conflicts that the editors consider relevant to the content of the manuscript have been disclosed.
