# Supplementary material for: High Risk of Plasmodium vivax Malaria Following Splenectomy in Papua, Indonesia
Source: Clin Infect Dis. 2018 May 16;68(1):51–60. doi: 10.1093/cid/ciy403 (PMC6128403; doi:10.1093/cid/ciy403)
Supplement: Supplementary Material [file ciy403_suppl_supplementary_materials.docx]

**Supplementary File 1.** Definitions for diagnosis of trauma

In the sensitivity analysis, the risks of splenectomy were compared to non-splenectomy trauma controls (a subgroup of the primary comparator group restricted to patients admitted to hospital for trauma). Patients admitted for trauma were determined by searching for diagnoses containing the following words: "trauma", "Trauma", "Traumatic", "fracture", "disloc","Disloc", "superficial injuries", "Superficial injuries", "Motorcycle", "injuries", "Injuries", "Contusion", "injury", "Injury", "Car occup", "Driver", "Pedestrian", "motor", "vehicle", "Unspecified fall".

**Supplementary Table 1.** Reason for splenectomy

| Reason for splenectomy | Number of patients (%) |
| --- | --- |
| Trauma | 50 (74.6) |
| Passenger in vehicle accident | 16 |
| Pedestrian in vehicle accident | 2 |
| Assault by sharp object | 4 |
| Assault by blunt object | 5 |
| Assault by bodily force | 20 |
| Unspecified fall | 3 |
| Non-trauma | 2 (3) |
| Unclear | 15 (22.4) |
| Total | 67 (100) |

**Supplementary Table 2.** Risk of representation to hospital with malaria, death, and admission with different clinical outcomes within 12 months of splenectomy versus non-splenectomy trauma controls

| **Outcomes Observed**  **within 12 Months** | **Frequency of Outcome (n/N)** | |  | **Splenectomy Patients VS Non-splenectomy Trauma Controls** | | | | | | |
| --- | --- | --- | --- | --- | --- | --- | --- | --- | --- | --- |
|  | **Splenectomy**  **Patients** | **Non-splenectomy**  **Trauma Controls** |  | **Univariable Analysis** | |  | **Multivariable Analysis ^a^** | |  |  |
|  |  |  |  | **HR [95% CI]** | ***P*** |  | **AHR [95% CI]** | ***P*** |  |  |
| Any malaria | 43.3% (29/67) | 8.2% (133/1,631) |  | 7.2 [4.8-10.7] | <0.001 |  | 6.1 [3.9-9.3] | <0.001 |  |  |
| *P. falciparum* | 17.9% (12/67) | 5.2% (84/1,631) |  | 3.8 [2.1-7.0] | <0.001 |  | 3.6 [1.9-6.8] | <0.001 |  |  |
| *P. vivax* | 31.3% (21/67) | 2.2% (36/1,631) |  | 17.1 [10.0-29.3] | <0.001 |  | 14.9 [8.2-27.0] | <0.001 |  |  |
| Death | 6.0% (4/67) | 3.7% (61/1,631) |  | 1.6 [0.6-4.4] | 0.373 |  | 1.4 [0.5-4.0] | 0.510 |  |  |
| Any hospital representation | 82.1% (55/67) | 59.3% (967/1,631) |  | 1.6 [1.2-2.1] | 0.001 |  | 1.6 [1.2-2.2] | 0.001 |  |  |
| Any hospital admission | 20.9% (14/67) | 8.6% (140/1,631) |  | 2.6 [1.5-4.5] | 0.001 |  | 2.5 [1.4-4.4] | 0.002 |  |  |
| Admission with pneumonia | 4.5% (3/67) | 0.2% (3/1,631) |  | 24.1 [4.9-119.6] | <0.001 |  | 33.5 [5.2-217.2] | <0.001 |  |  |
| Admission with diarrhea | 6.0% (4/67) | 0.4% (6/1,631) |  | 16.8 [4.7-59.4] | <0.001 |  | 7.7 [1.8-33.6] | 0.006 |  |  |
| Admission with sepsis | 1.5% (1/67) | 0.3% (5/1,631) |  | 4.9 [0.6-41.8] | 0.148 |  | 5.6 [0.6-50.6] | 0.128 |  |  |
| Admission with cellulitis | 0% (0/67) | 0.2% (4/1,631) |  | 0.0 | 0.787 |  | - | - |  |  |
| Admission with tuberculosis | 0% (0/67) | 0.5% (8/1,631) |  | 0.0 | 0.703 |  | - | - |  |  |
| Admission with UTI | 3.0% (2/67) | 0.7% (11/1,631) |  | 4.4 [1.0-19.9] | 0.054 |  | 3.4 [0.7-15.9] | 0.116 |  |  |

Abbreviations: HR=Hazard Risk; AHR=Adjusted Hazard Risk; CI=Confidence Interval; UTI, Urinary Tract Infection.

^a^ Separate Cox model generated for each outcome, included co-variables age, gender, ethnicity (non-Papuan, highland-Papuan, lowland-Papuan) and peripheral parasitemia (negative, *P. falciparum*, *P. vivax*, mixed infections) stratified by year of splenectomy or 1^st^ hospital admission (2004-2013).

**Supplementary Table 3.** Characteristics of 11 splenectomized patients followed prospectively

| Variable | | Splenectomy Patients |
| --- | --- | --- |
|  | | Number (%) |
| Gender | |  |
| Male | | 9 (81.8) |
| Female | | 2 (18.2) |
|  | |  |
| Ethnicity | |  |
| Non-Papuan | | 3 (27.3) |
| Highland-Papuan | | 6 (54.5) |
| Lowland-Papuan | | 2 (18.2) |
|  | |  |
| Age (years) | |  |
| Median [Range] | | 30 [15-46] |
|  | |  |
| Parasitemia during surgery |  |  |
| Negative | | 7 (63.6) |
| *P. falciparum* | | 3 (27.3) |
| *P. vivax* | | 1 (9.1) |
|  | |  |
| Patients with malaria within 14 months | |  |
| None | | 3 (27.3) |
| *P. falciparum* only | | 0 |
| *P. vivax* only | | 3 (27.3) |
| Both species | | 5 (45.4) |
|  | |  |
| Total | | 11 (100) |

**Supplementary Table 4.** Risk factors at initial presentation for representing with any malaria, *P. falciparum*, or *P. vivax* within 4 years

| **Outcome within 4 years** | **Baseline Risk Factor** |  | **Prevalence of Outcome (n/N)** |  | **Univariable Analysis** | |  | **Multivariable Analysis ^a^** | |
| --- | --- | --- | --- | --- | --- | --- | --- | --- | --- |
|  |  |  |  |  | **HR [95% CI]** | ***P*** |  | **AHR [95% CI]** | ***P*** |
| **Any malaria** | **Splenectomy** |  |  |  |  |  |  |  |  |
|  | No |  | 20.3% (2,173/10,707) |  | Reference |  |  | Reference |  |
|  | Yes |  | 50.7% (34/67) |  | 3.6 [.6-5.1] | <0.001 |  | 4.4 [3.1-6.3] | <0.001 |
|  | **Gender** |  |  |  |  |  |  |  |  |
|  | Male |  | 18.6% (1,229/6,601) |  | Reference |  |  | Reference |  |
|  | Female |  | 23.4% (978/4,173) |  | 1.3 [1.2-1.4] | <0.001 |  | 1.3 [1.2-1.4] | <0.001 |
|  | **Age (years)** |  |  |  |  |  |  |  |  |
|  | ≥15 to 60 |  | 20.1% (2,057/10,230) |  | Reference |  |  | Reference |  |
|  | 12 to 14 |  | 27.6% (150/544) |  | 1.4 [1.2-1.6] | <0.001 |  | 1.2 [1.0-1.4] | 0.066 |
|  | **Ethnicity** |  |  |  |  |  |  |  |  |
|  | Non-Papuan |  | 8.9% (245/2,764) |  | Reference |  |  | Reference |  |
|  | Highland-Papuan |  | 28.7% (1,809/6,306) |  | 3.3 [2.9-3.8] | <0.001 |  | 3.2 [2.9-3.7] | <0.001 |
|  | Lowland-Papuan |  | 9.0% (152/1,682) |  | 1.0 [0.8-1.2] | 0.771 |  | 1.1 [0.9-1.4] | 0.267 |
|  | **Parasitemia** |  |  |  |  |  |  |  |  |
|  | Negative |  | 14.7% (883/5,990) |  | Reference |  |  | Reference |  |
|  | *P. falciparum* |  | 27.3% (900/3,301) |  | 1.8 [1.6-2.0] | <0.001 |  | 1.6 [1.5-1.8] | <0.001 |
|  | *P. vivax* |  | 27.1% (221/814) |  | 1.9 [1.6-2.2] | <0.001 |  | 2.0 [1.7-2.3] | <0.001 |
|  | Mixed infections |  | 31.1% (190/611) |  | 2.2 [1.9-2.6] | <0.001 |  | 1.9 [1.6-2.2] | <0.001 |
| ***P. falciparum*** | **Splenectomy** |  |  |  |  |  |  |  |  |
|  | No |  | 14.2% (1,525/10,707) |  | Reference |  |  | Reference |  |
|  | Yes |  | 25.4% (17/67) |  | 2.1 [1.3-3.4] | 0.002 |  | 2.8 [1.7-4.5] | <0.001 |
|  | **Gender** |  |  |  |  |  |  |  |  |
|  | Male |  | 12.9% (850/6,601) |  | Reference |  |  | Reference |  |
|  | Female |  | 16.6% (692/4,173) |  | 1.3 [1.2-1.5] | <0.001 |  | 1.3 [1.2-1.4] | <0.001 |
|  | **Age (years)** |  |  |  |  |  |  |  |  |
|  | ≥15 to 60 |  | 14.0% (1,434/10,230) |  | Reference |  |  | Reference |  |
|  | 12 to 14 |  | 19.9% (108/544) |  | 1.4 [1.1-1.7] | 0.002 |  | 1.2 [1.0-1.4] | 0.136 |
|  | **Ethnicity** |  |  |  |  |  |  |  |  |
|  | Non-Papuan |  | 5.4% (148/2,764) |  | Reference |  |  | Reference |  |
|  | Highland-Papuan |  | 20.3% (1,277/6,306) |  | 3.7 [3.1-4.4] | <0.001 |  | 3.4 [2.8-4.0] | <0.001 |
|  | Lowland-Papuan |  | 6.9% (116/1,682) |  | 1.2 [1.0-1.6] | 0.089 |  | 1.4 [1.1-1.8] | 0.014 |
|  | **Parasitemia** |  |  |  |  |  |  |  |  |
|  | Negative |  | 10.2% (611/5,990) |  | Reference |  |  | Reference |  |
|  | *P. falciparum* |  | 20.4% (672/3,301) |  | 1.9 [1.7-2.1] | <0.001 |  | 1.6 [1.5-1.8] | <0.001 |
|  | *P. vivax* |  | 17.1% (139/814) |  | 1.6 [1.3-1.9] | <0.001 |  | 1.7 [1.4-2.0] | <0.001 |
|  | Mixed infections |  | 18.0% (110/611) |  | 1.7 [1.4-2.1] | <0.001 |  | 1.5 [1.2-1.8] | <0.001 |
| ***P. vivax*** | **Splenectomy** |  |  |  |  |  |  |  |  |
|  | No |  | 7.6% (810/10,707) |  | Reference |  |  | Reference |  |
|  | Yes |  | 38.8% (26/67) |  | 6.8 [4.6-10.0] | <0.001 |  | 7.3 [4.9-10.9] | <0.001 |
|  | **Gender** |  |  |  |  |  |  |  |  |
|  | Male |  | 6.6% (437/6,601) |  | Reference |  |  | Reference |  |
|  | Female |  | 9.6% (399/4,173) |  | 1.5 [1.3-1.7] | <0.001 |  | 1.4 [1.2-1.6] | <0.001 |
|  | **Age (years)** |  |  |  |  |  |  |  |  |
|  | ≥15 to 60 |  | 7.5% (771/10,230) |  | Reference |  |  | Reference |  |
|  | 12 to 14 |  | 11.9% (65/544) |  | 1.5 [1.2-2.0] | 0.001 |  | 1.4 [1.1-1.8] | 0.019 |
|  | **Ethnicity** |  |  |  |  |  |  |  |  |
|  | Non-Papuan |  | 4.1% (114/2,764) |  | Reference |  |  | Reference |  |
|  | Highland-Papuan |  | 10.9% (690/6,306) |  | 2.5 [2.0-3.0] | <0.001 |  | 2.6 [2.1-3.2] | <0.001 |
|  | Lowland-Papuan |  | 1.9% (32/1,682) |  | 0.4 [0.3-0.6] | <0.001 |  | 0.6 [0.4-0.8] | 0.003 |
|  | **Parasitemia** |  |  |  |  |  |  |  |  |
|  | Negative |  | 4.8% (288/5,990) |  | Reference |  |  | Reference |  |
|  | *P. falciparum* |  | 10.3% (340/3,301) |  | 2.0 [1.7-2.3] | <0.001 |  | 1.8 [1.6-2.2] | <0.001 |
|  | *P. vivax* |  | 14.9% (121/814) |  | 3.0 [2.4-3.7] | <0.001 |  | 3.0 [2.4-3.7] | <0.001 |
|  | Mixed infections |  | 13.6% (83/611) |  | 2.8 [2.2-3.5] | <0.001 |  | 2.3 [1.8-2.9] | <0.001 |

Abbreviations: HR=Hazard Risk; AHR=Adjusted Hazard Risk; CI=Confidence Interval.

^a^ Cox model stratified by year of splenectomy or 1^st^ hospital admission (2004-2013).

**Supplementary Table 5.** Risk factors at initial presentation for dying within 12 months

| **Baseline Risk Factors** |  | **Prevalence of death (n/N)** |  | **Univariable Analysis** | |  | **Multivariable Analysis ^a^** | |
| --- | --- | --- | --- | --- | --- | --- | --- | --- |
|  |  |  |  | **HR [95% CI]** | ***P*** |  | **AHR [95% CI]** | ***P*** |
| **Splenectomy** |  |  |  |  |  |  |  |  |
| No |  | 5.7% (613/10,707) |  | Reference |  |  | Reference |  |
| Yes |  | 6.0% (4/67) |  | 1.0 [0.4-2.8] | 0.951 |  | 0.8 [0.3-2.2] | 0.671 |
| **Gender** |  |  |  |  |  |  |  |  |
| Male |  | 6.1% (402/6,601) |  | Reference |  |  | Reference |  |
| Female |  | 5.2% (215/4,173) |  | 0.8 [0.7-1.0] | 0.042 |  | 0.9 [0.7-1.0] | 0.059 |
| **Age (years)** |  |  |  |  |  |  |  |  |
| ≥15 to 60 |  | 5.9% (604/10,230) |  | Reference |  |  | Reference |  |
| 12 to 14 |  | 2.4% (13/544) |  | 0.4 [0.2-0.7] | 0.001 |  | 0.4 [0.3-0.8] | 0.003 |
| **Ethnicity** |  |  |  |  |  |  |  |  |
| Non-Papuan |  | 5.9% (162/2,764) |  | Reference |  |  | Reference |  |
| Highland-Papuan |  | 5.2% (328/6,306) |  | 0.9 [0.7-1.1] | 0.164 |  | 0.9 [0.8-1.1] | 0.543 |
| Lowland-Papuan |  | 7.5% (126/1,682) |  | 1.3 [1.0-1.6] | 0.037 |  | 1.1 [0.9-1.4] | 0.419 |
| **Parasitemia** |  |  |  |  |  |  |  |  |
| Negative |  | 7.5% (447/5,990) |  | Reference |  |  | Reference |  |
| *P. falciparum* |  | 3.7% (122/3,301) |  | 0.5 [0.4-0.6] | <0.001 |  | 0.5 [0.4-0.6] | <0.001 |
| *P. vivax* |  | 2.9% (24/814) |  | 0.4 [0.3-0.6] | <0.001 |  | 0.4 [0.3-0.6] | <0.001 |
| Mixed infections |  | 5.7% (20/611) |  | 0.4 [0.3-0.7] | <0.001 |  | 0.4 [0.3-0.7] | <0.001 |

Abbreviations: HR=Hazard Risk; AHR=Adjusted Hazard Risk; CI=Confidence Interval.

^a^ Cox model stratified by year of splenectomy or 1^st^ hospital admission (2004-2013).

**Supplementary Table 6.**  Risk factors at initial presentation for representing to hospital within 12 months

| **Baseline Risk Factors** |  | **Prevalence of representing to hospital (n/N)** |  | **Univariable Analysis** | |  | **Multivariable Analysis ^a^** | |
| --- | --- | --- | --- | --- | --- | --- | --- | --- |
|  |  |  |  | **HR [95% CI]** | ***P*** |  | **AHR [95% CI]** | ***P*** |
| **Splenectomy** |  |  |  |  |  |  |  |  |
| No |  | 48.1% (5,153/10,707) |  | Reference |  |  | Reference |  |
| Yes |  | 82.1% (55/67) |  | 2.7 [2.1-3.6] | <0.001 |  | 2.3 [1.8-3.0] | <0.001 |
| **Gender** |  |  |  |  |  |  |  |  |
| Male |  | 46.6% (3,077/6,601) |  | Reference |  |  | Reference |  |
| Female |  | 51.1% (2,131/4,173) |  | 1.1 [1.1-1.2] | <0.001 |  | 1.1 [1.0-1.2] | 0.001 |
| **Age (years)** |  |  |  |  |  |  |  |  |
| ≥15 to 60 |  | 48.4% (4,953/10,230) |  | Reference |  |  | Reference |  |
| 12 to 14 |  | 46.9% (255/544) |  | 0.9 [0.8-1.0] | 0.123 |  | 0.9 [0.8-1.0] | 0.164 |
| **Ethnicity** |  |  |  |  |  |  |  |  |
| Non-Papuan |  | 38.7% (1,069/2,764) |  | Reference |  |  | Reference |  |
| Highland-Papuan |  | 51.8% (3,266/6,306) |  | 1.3 [1.2-1.4] | <0.001 |  | 1.3 [1.2-1.4] | <0.001 |
| Lowland-Papuan |  | 51.4% (864/1,682) |  | 1.5 [1.3-1.6] | <0.001 |  | 1.2 [1.1-1.4] | <0.001 |
| **Parasitemia** |  |  |  |  |  |  |  |  |
| Negative |  | 53.6% (3,213/5,990) |  | Reference |  |  | Reference |  |
| *P. falciparum* |  | 40.8% (1,348/3,301) |  | 0.60 [0.56-0.64] | <0.001 |  | 0.61 [0.57-0.66] | <0.001 |
| *P. vivax* |  | 44.8% (365/814) |  | 0.69 [0.62-0.76] | <0.001 |  | 0.72 [0.65-0.80] | <0.001 |
| Mixed infections |  | 41.9% (256/611) |  | 0.63 [0.55-0.71] | <0.001 |  | 0.63 [0.55-0.71] | <0.001 |

Abbreviations: HR=Hazard Risk; AHR=Adjusted Hazard Risk; CI=Confidence Interval.

^a^ Cox model stratified by year of splenectomy or 1^st^ hospital admission (2004-2013).

**Supplementary Table 7.**  Risk factors at initial presentation for admission to hospital within 12 months

| **Baseline Risk Factors** |  | **Prevalence of hospital admission (n/N)** |  | **Univariable Analysis** | |  | **Multivariable Analysis ^a^** | |
| --- | --- | --- | --- | --- | --- | --- | --- | --- |
|  |  |  |  | **HR [95% CI]** | ***P*** |  | **AHR [95% CI]** | ***P*** |
| **Splenectomy** |  |  |  |  |  |  |  |  |
| No |  | 12.2% (1,308/10,707) |  | Reference |  |  | Reference |  |
| Yes |  | 20.9% (14/67) |  | 1.8 [1.1-3.1] | 0.027 |  | 1.8 [1.0-3.0] | 0.037 |
| **Gender** |  |  |  |  |  |  |  |  |
| Male |  | 10.9% (721/6,601) |  | Reference |  |  | Reference |  |
| Female |  | 14.4% (601/4,173) |  | 1.3 [1.2-1.5] | <0.001 |  | 1.3 [1.2-1.5] | <0.001 |
| **Age (years)** |  |  |  |  |  |  |  |  |
| ≥15 to 60 |  | 12.4% (1,264/10,230) |  | Reference |  |  | Reference |  |
| 12 to 14 |  | 10.7% (58/544) |  | 0.8 [0.6-1.1] | 0.132 |  | 0.8 [0.6-1.0] | 0.097 |
| **Ethnicity** |  |  |  |  |  |  |  |  |
| Non-Papuan |  | 8.1% (224/2,764) |  | Reference |  |  | Reference |  |
| Highland-Papuan |  | 14.7% (926/6,306) |  | 1.8 [1.5-2.0] | <0.001 |  | 1.7 [1.4-2.0] | <0.001 |
| Lowland-Papuan |  | 10.2% (172/1,682) |  | 1.2 [1.0-1.5] | 0.030 |  | 1.1 [0.9-1.4] | 0.297 |
| **Parasitemia** |  |  |  |  |  |  |  |  |
| Negative |  | 13.1% (786/5,990) |  | Reference |  |  | Reference |  |
| *P. falciparum* |  | 10.4% (342/3,301) |  | 0.7 [0.6-0.8] | <0.001 |  | 0.7 [0.6-0.8] | <0.001 |
| *P. vivax* |  | 13.8% (112/814) |  | 1.0 [0.8-1.2] | 0.955 |  | 1.0 [0.8-1.2] | 0.921 |
| Mixed infections |  | 11.6% (71/611) |  | 0.8 [0.7-1.1] | 0.137 |  | 0.8 [0.6-1.0] | 0.772 |

Abbreviations: HR=Hazard Risk; AHR=Adjusted Hazard Risk; CI=Confidence Interval.

^a^ Cox model stratified by year of splenectomy or 1^st^ hospital admission (2004-2013).

**Supplementary Table 8.**  Risk factors at initial presentation for being admitted with pneumonia within 12 months

| **Baseline Risk Factors** |  | **Prevalence of pneumonia (n/N)** |  | **Univariable Analysis** | |  | **Multivariable Analysis** **^a^** | |
| --- | --- | --- | --- | --- | --- | --- | --- | --- |
|  |  |  |  | **HR [95% CI]** | ***P*** |  | **AHR [95% CI]** | ***P*** |
| **Splenectomy** |  |  |  |  |  |  |  |  |
| No |  | 1.3% (138/10,707) |  | Reference |  |  | Reference |  |
| Yes |  | 4.5% (3/67) |  | 3.4 [1.1-10.7] | 0.036 |  | 2.8 [0.9-8.8] | 0.085 |
| **Gender** |  |  |  |  |  |  |  |  |
| Male |  | 1.2% (79/6,601) |  | Reference |  |  | Reference |  |
| Female |  | 1.5% (62/4,173) |  | 1.2 [0.9-1.7] | 0.196 |  | 1.2 [0.9-1.7] | 0.217 |
| **Age (years)** |  |  |  |  |  |  |  |  |
| ≥15 to 60 |  | 1.3% (135/10,230) |  | Reference |  |  | Reference |  |
| 12 to 14 |  | 1.1% (6/544) |  | 0.8 [0.4-1.8] | 0.583 |  | 0.8 [0.4-1.8] | 0.592 |
| **Ethnicity** |  |  |  |  |  |  |  |  |
| Non-Papuan |  | 0.4% (12/2,764) |  | Reference |  |  | Reference |  |
| Highland-Papuan |  | 1.8% (115/6,306) |  | 4.0 [2.6-7.3] | <0.001 |  | 4.2 [2.3-7.8] | <0.001 |
| Lowland-Papuan |  | 0.8% (14/1,682) |  | 1.8 [0.9-4.1] | 0.108 |  | 1.6 [0.7-3.5] | 0.252 |
| **Parasitemia** |  |  |  |  |  |  |  |  |
| Negative |  | 1.7% (100/5,990) |  | Reference |  |  | Reference |  |
| *P. falciparum* |  | 0.9% (29/3,301) |  | 0.5 [0.3-0.7] | 0.001 |  | 0.4 [0.3-0.6] | <0.001 |
| *P. vivax* |  | 0.9% (7/814) |  | 0.5 [0.2-1.0] | 0.065 |  | 0.5 [0.2-1.1] | 0.082 |
| Mixed infections |  | 0.7% (4/611) |  | 0.4 [0.1-1.0] | 0.051 |  | 0.3 [0.1-0.8] | 0.020 |

Abbreviations: HR=Hazard Risk; AHR=Adjusted Hazard Risk; CI=Confidence Interval.

^a^ Cox model stratified by year of splenectomy or 1^st^ hospital admission (2004-2013).

**Supplementary Table 9.**  Risk factors at initial presentation for being admitted with diarrhea within 12 months

| **Baseline Risk Factors** |  | **Prevalence of diarrhea (n/N)** |  | **Univariable Analysis** | |  | **Multivariable Analysis** **^a^** | |
| --- | --- | --- | --- | --- | --- | --- | --- | --- |
|  |  |  |  | **HR [95% CI]** | ***P*** |  | **AHR [95% CI]** | ***P*** |
| **Splenectomy** |  |  |  |  |  |  |  |  |
| No |  | 1.5% (160/10,707) |  | Reference |  |  | Reference |  |
| Yes |  | 6.0% (4/67) |  | 4.1 [1.5-11.0] | 0.006 |  | 3.5 [1.3-9.6] | 0.016 |
| **Gender** |  |  |  |  |  |  |  |  |
| Male |  | 1.3% (89/6,601) |  | Reference |  |  | Reference |  |
| Female |  | 1.8% (75/4,173) |  | 1.3 [1.0-1.8] | 0.060 |  | 1.3 [1.0-1.8] | 0.094 |
| **Age (years)** |  |  |  |  |  |  |  |  |
| ≥15 to 60 |  | 1.6% (161/10,230) |  | Reference |  |  | Reference |  |
| 12 to 14 |  | 0.6% (3/544) |  | 0.3 [0.1-1.0] | 0.058 |  | 0.3 [0.1-1.0] | 0.043 |
| **Ethnicity** |  |  |  |  |  |  |  |  |
| Non-Papuan |  | 0.4% (10/2,764) |  | Reference |  |  | Reference |  |
| Highland-Papuan |  | 3.3% (144/6,306) |  | 6.0 [3.2-11.4] | <0.001 |  | 6.3 [3.3-12.1] | <0.001 |
| Lowland-Papuan |  | 0.6% (10/1,682) |  | 1.6 [0.7-3.9] | 0.287 |  | 1.5 [0.6-3.7] | 0.350 |
| **Parasitemia** |  |  |  |  |  |  |  |  |
| Negative |  | 1.6% (96/5,990) |  | Reference |  |  | Reference |  |
| *P. falciparum* |  | 1.5% (48/3,301) |  | 0.8 [0.6-1.2] | 0.339 |  | 0.7 [0.5-1.1] | 0.103 |
| *P. vivax* |  | 1.5% (12/814) |  | 0.9 [0.5-1.6] | 0.652 |  | 0.9 [0.5-1.7] | 0.793 |
| Mixed infections |  | 1.0% (6/611) |  | 0.6 [0.3-1.3] | 0.193 |  | 0.5 [0.2-1.1] | 0.078 |

Abbreviations: HR=Hazard Risk; AHR=Adjusted Hazard Risk; CI=Confidence Interval.

^a^ Cox model stratified by year of splenectomy or 1^st^ hospital admission (2004-2013).

**Supplementary Table 10.** Risk factors at initial presentation for being admitted with Sepsis within 12 months

| **Baseline Risk Factors** |  | **Prevalence of sepsis (n/N)** |  | **Univariable Analysis** | |  | **Multivariable Analysis** **^a^** | |
| --- | --- | --- | --- | --- | --- | --- | --- | --- |
|  |  |  |  | **HR [95% CI]** | ***P*** |  | **AHR [95% CI]** | ***P*** |
| **Splenectomy** |  |  |  |  |  |  |  |  |
| No |  | 0.4% (47/10,707) |  | Reference |  |  | Reference |  |
| Yes |  | 1.5% (1/67) |  | 3.4 [0.5-24.4] | 0.230 |  | 2.6 [0.4-19.3] | 0.350 |
| **Gender** |  |  |  |  |  |  |  |  |
| Male |  | 0.4% (29/6,601) |  | Reference |  |  | Reference |  |
| Female |  | 0.5% (19/4,173) |  | 1.0 [0.6-1.8] | 0.910 |  | 1.1 [0.6-1.9] | 0.784 |
| **Age (years)** |  |  |  |  |  |  |  |  |
| ≥15 to 60 |  | 0.5% (48/10,230) |  | Reference |  |  | Reference |  |
| 12 to 14 |  | 0% (0/544) |  | 0.05 [0-12.6] | 0.282 |  | 0.0 | 0.963 |
| **Ethnicity** |  |  |  |  |  |  |  |  |
| Non-Papuan |  | 0.1% (4/2,764) |  | Reference |  |  | Reference |  |
| Highland-Papuan |  | 0.6% (36/6,306) |  | 3.8 [1.4-10.7] | 0.011 |  | 4.1 [1.4-11.9] | 0.009 |
| Lowland-Papuan |  | 0.5% (8/1,682) |  | 3.3 [1.0-10.9] | 0.053 |  | 2.9 [0.8-9.7] | 0.093 |
| **Parasitemia** |  |  |  |  |  |  |  |  |
| Negative |  | 0.6% (37/5,990) |  | Reference |  |  | Reference |  |
| *P. falciparum* |  | 0.2% (5/3,301) |  | 0.2 [0.1-0.6] | 0.002 |  | 0.2 [0.1-0.6] | 0.002 |
| *P. vivax* |  | 0.1% (1/814) |  | 0.2 [0.1-1.4] | 0.101 |  | 0.2 [0.1-1.6] | 0.134 |
| Mixed infections |  | 0.8% (5/611) |  | 1.3 [0.5-3.2] | 0.621 |  | 1.2 [0.5-3.1] | 0.350 |

Abbreviations: HR=Hazard Risk; AHR=Adjusted Hazard Risk; CI=Confidence Interval.

^a^ Cox model stratified by year of splenectomy or 1^st^ hospital admission (2004-2013).

**Supplementary Table 11.**  Risk factors at initial presentation for being admitted with cellulitis within 12 months

| **Baseline Risk Factors** |  | **Prevalence of cellulitis (n/N)** |  | **Univariable Analysis** | |  | **Multivariable Analysis** **^a^** | |
| --- | --- | --- | --- | --- | --- | --- | --- | --- |
|  |  |  |  | **HR [95% CI]** | ***P*** |  | **AHR [95% CI]** | ***P*** |
| **Splenectomy** |  |  |  |  |  |  |  |  |
| No |  | 0.1% (13/10,707) |  | Reference |  |  | Reference |  |
| Yes |  | 0% (0/67) |  | 0.0 | 0.848 |  | - | - |
| **Gender** |  |  |  |  |  |  |  |  |
| Male |  | 0.1% (9/6,601) |  | Reference |  |  | Reference |  |
| Female |  | 0.1% (4/4,173) |  | 0.7 [0.2-2.3] | 0.563 |  | 0.7 [0.2-2.3] | 0.568 |
| **Age (years)** |  |  |  |  |  |  |  |  |
| ≥15 to 60 |  | 0.1% (13/10,230) |  | Reference |  |  | Reference |  |
| 12 to 14 |  | 0% (0/544) |  | 0.0 | 0.574 |  | - | - |
| **Ethnicity** |  |  |  |  |  |  |  |  |
| Non-Papuan |  | 0.04% (1/2,764) |  | Reference |  |  | Reference |  |
| Highland-Papuan |  | 0.2% (12/6,306) |  | 5.0 [0.6-38.2] | 0.124 |  | 6.7 [0.8-55.8] | 0.079 |
| Lowland-Papuan |  | 0% (0/1,682) |  | 0.0 | 0.982 |  | - | - |
| **Parasitemia** |  |  |  |  |  |  |  |  |
| Negative |  | 0.1% (8/5,990) |  | Reference |  |  | Reference |  |
| *P. falciparum* |  | 0.1% (4/3,301) |  | 0.8 [0.3-2.8] | 0.785 |  | 0.8 [0.2-2.7] | 0.730 |
| *P. vivax* |  | 0.1% (1/814) |  | 0.9 [0.1-7.0] | 0.897 |  | 1.1 [0.1-8.6] | 0.960 |
| Mixed infections |  | 0% (0/611) |  | 0.0 | 0.979 |  | - | - |

Abbreviations: HR=Hazard Risk; AHR=Adjusted Hazard Risk; CI=Confidence Interval.

^a^ Cox model stratified by year of splenectomy or 1^st^ hospital admission (2004-2013).

**Supplementary Table 12.**  Risk factors at initial presentation for being admitted with tuberculosis within 12 months

| **Baseline Risk Factors** |  | **Prevalence of tuberculosis (n/N)** |  | **Univariable Analysis** | |  | **Multivariable Analysis** **^a^** | |
| --- | --- | --- | --- | --- | --- | --- | --- | --- |
|  |  |  |  | **HR [95% CI]** | ***P*** |  | **AHR [95% CI]** | ***P*** |
| **Splenectomy** |  |  |  |  |  |  |  |  |
| No |  | 2.5% (269/10,707) |  | Reference |  |  | Reference |  |
| Yes |  | 0% (0/67) |  | 0.0 | 0.380 |  | - | - |
| **Gender** |  |  |  |  |  |  |  |  |
| Male |  | 2.5% (162/6,601) |  | Reference |  |  | Reference |  |
| Female |  | 2.6% (107/4,173) |  | 1.0 [0.8-1.3] | 0.725 |  | 1.0 [0.8-1.3] | 0.716 |
| **Age (years)** |  |  |  |  |  |  |  |  |
| ≥15 to 60 |  | 2.6% (264/10,230) |  | Reference |  |  | Reference |  |
| 12 to 14 |  | 0.9% (5/544) |  | 0.3 [0.1-0.8] | 0.016 |  | 0.3 [0.1-0.8] | 0.016 |
| **Ethnicity** |  |  |  |  |  |  |  |  |
| Non-Papuan |  | 0.8% (22/2,764) |  | Reference |  |  | Reference |  |
| Highland-Papuan |  | 3.3% (206/6,306) |  | 4.0 [2.5-6.1] | <0.001 |  | 4.4 [2.8-6.8] | <0.001 |
| Lowland-Papuan |  | 2.4% (41/1,682) |  | 3.0 [1.8-5.1] | <0.001 |  | 2.4 [1.4-4.1] | 0.001 |
| **Parasitemia** |  |  |  |  |  |  |  |  |
| Negative |  | 3.5% (212/5,990) |  | Reference |  |  | Reference |  |
| *P. falciparum* |  | 1.0% (32/3,301) |  | 0.3 [0.2-0.4] | <0.001 |  | 0.2 [0.2-0.3] | <0.001 |
| *P. vivax* |  | 2.3% (19/814) |  | 0.6 [0.4-1.0] | 0.049 |  | 0.7 [0.4-1.1] | 0.104 |
| Mixed infections |  | 0.8% (5/611) |  | 0.2 [0.1-0.5] | 0.001 |  | 0.2 [0.1-0.5] | <0.001 |

Abbreviations: HR=Hazard Risk; AHR=Adjusted Hazard Risk; CI=Confidence Interval.

^a^ Cox model stratified by year of splenectomy or 1^st^ hospital admission (2004-2013).

**Supplementary Table 13.**  Risk factors at initial presentation for being admitted with urinary tract infection within 12 months

| **Baseline Risk Factors** |  | **Prevalence of UTI (n/N)** |  | **Univariable Analysis** | |  | **Multivariable Analysis** **^a^** | |
| --- | --- | --- | --- | --- | --- | --- | --- | --- |
|  |  |  |  | **HR [95% CI]** | ***P*** |  | **AHR [95% CI]** | ***P*** |
| **Splenectomy** |  |  |  |  |  |  |  |  |
| No |  | 1.3% (139/10,707) |  | Reference |  |  | Reference |  |
| Yes |  | 3.0% (2/67) |  | 2.3 [0.6-9.2] | 0.250 |  | 2.3 [0.6-9.2] | 0.256 |
| **Gender** |  |  |  |  |  |  |  |  |
| Male |  | 0.8% (56/6,601) |  | Reference |  |  | Reference |  |
| Female |  | 2.0% (85/4,173) |  | 2.4 [1.7-3.4] | <0.001 |  | 2.5 [1.8-3.6] | <0.001 |
| **Age (years)** |  |  |  |  |  |  |  |  |
| ≥15 to 60 |  | 1.3% (134/10,230) |  | Reference |  |  | Reference |  |
| 12 to 14 |  | 1.3% (7/544) |  | 0.9 [0.4-2.0] | 0.862 |  | 0.9 [0.4-1.9] | 0.711 |
| **Ethnicity** |  |  |  |  |  |  |  |  |
| Non-Papuan |  | 1.0% (28/2,764) |  | Reference |  |  | Reference |  |
| Highland-Papuan |  | 1.6% (103/6,306) |  | 1.5 [1.0-2.3] | 0.048 |  | 1.6 [1.0-2.4] | 0.045 |
| Lowland-Papuan |  | 0.6% (10/1,682) |  | 0.6 [0.3-1.2] | 0.132 |  | 0.5 [0.3-1.1] | 0.104 |
| **Parasitemia** |  |  |  |  |  |  |  |  |
| Negative |  | 1.3% (79/5,990) |  | Reference |  |  | Reference |  |
| *P. falciparum* |  | 1.2% (39/3,301) |  | 0.8 [0.6-1.2] | 0.362 |  | 0.7 [0.5-1.0] | 0.063 |
| *P. vivax* |  | 1.7% (14/814) |  | 1.2 [0.7-2.2] | 0.457 |  | 1.0 [0.6-1.8] | 0.966 |
| Mixed infections |  | 1.5% (9/611) |  | 1.1 [0.5-2.1] | 0.876 |  | 0.9 [0.5-1.9] | 0.861 |

Abbreviations: HR=Hazard Risk; AHR=Adjusted Hazard Risk; CI=Confidence Interval; UTI=Urinary Tract Infection.

^a^ Cox model stratified by year of splenectomy or 1^st^ hospital admission (2004-2013).
